# Generalized spring tensor models for protein fluctuation dynamics and conformation changes

**DOI:** 10.1186/1472-6807-10-S1-S3

**Published:** 2010-05-17

**Authors:** Tu-Liang Lin, Guang Song

**Affiliations:** 1 Computer Science Department, Iowa State University, 226 Atanasoff Hall, Ames, IA 50011, USA; 2 L H Baker Center for Bioinformatics and Biological Statistics, Iowa State University, Ames, IA 50011, USA; 3 Program of Bioinformatics and Computational Biology, Iowa State University, Ames, IA 50011, USA

## Abstract

**Background:**

In the last decade, various coarse-grained elastic network models have been developed to study the large-scale motions of proteins and protein complexes where computer simulations using detailed all-atom models are not feasible. Among these models, the Gaussian Network Model (GNM) and Anisotropic Network Model (ANM) have been widely used. Both models have strengths and limitations. GNM can predict the relative magnitudes of protein fluctuations well, but due to its isotropy assumption, it can not be applied to predict the directions of the fluctuations. In contrast, ANM adds the ability to do the latter, but loses a significant amount of precision in the prediction of the magnitudes.

**Results:**

In this article, we develop a single model, called generalized spring tensor model (STeM), that is able to predict well both the magnitudes and the directions of the fluctuations. Specifically, STeM performs equally well in B-factor predictions as GNM* and* has the ability to predict the directions of fluctuations as ANM. This is achieved by employing a physically more realistic potential, the G*ō*-like potential. The potential, which is more sophisticated than that of either GNM or ANM, though adds complexity to the derivation process of the Hessian matrix (which fortunately has been done once for all and the MATLAB code is freely available electronically at http://www.cs.iastate.edu/~gsong/STeM), causes virtually no performance slowdown.

**Conclusions:**

Derived from a physically more realistic potential, STeM proves to be a natural solution in which advantages that used to exist in two separate models, namely GNM and ANM, are achieved in one single model. It thus lightens the burden to work with two separate models and to relate the modes of GNM with those of ANM at times. By examining the contributions of different interaction terms in the G*ō* potential to the fluctuation dynamics, STeM reveals, (i) a physical explanation for why the distance-dependent, inverse distance square (i.e., ) spring constants perform better than the uniform ones, and (ii), the importance of three-body and four-body interactions to properly modeling protein dynamics.

## Introduction

It is now well accepted that the functions of a protein are closely related to not only its structure but also its dynamics. With the advancement of the computational power and increasing availability of computational resources, function-related protein dynamics, such as large-scale conformation transitions, has been probed by various computational methods at multiple scales. Among these computational methods, coarse-grained models play an important role since many functional processes take place over time scales that are well beyond the capacity of all-atom simulations [1]. One type of coarse-grained models, the elastic network models (ENMs), have been particularly successful and widely used in studying protein dynamics and in relating the intrinsic motions of a protein with its functional-related conformation changes over the last decade [2-5].

The reason why ENMs have been well received as compared to the conventional normal mode analysis (NMA) lies at its simplicity to use. ENMs do not require energy minimization and therefore can be applied directly to crystal structures to compute the modes of motions. In contrast, minimization is required for carrying out the conventional normal mode analysis (NMA). The problematic aspect of energy minimization is that it usually shifts the protein molecule away from its crystal conformation by about 2 Å. In addition, in ENMs analytical solutions to residue fluctuations and motion correlations can be easily derived. On the other hand, the simplicity of ENMs leaves much room for improvement and many new models have been proposed [6-12].

The two most widely used ENM models are Gaussian Network Model (GNM) and Anisotropic Network Model (ANM). They have been used to predict the magnitudes or directions of the residue fluctuations from a single structure and have been applied in many research areas [4,5], such as domain decomposition [13] and allosteric communication [14-17]. Both models have their own advantages and disadvantages. GNM can predict the relative magnitudes of the fluctuations well, but due to its isotropy assumption, it can not be applied to predict the directions of the fluctuations. In contrast, ANM adds the ability to do the latter, but it loses a significant amount of precision in the prediction of the magnitudes.

**Gaussian network model.** Gaussian Network Model (GNM) was first introduced in [2] under the assumption that the separation between a pair of residues in the folded protein is Gaussianly distributed. Given its simplicity, the model performs extremely well in predicting the experimental B-factors. The model represents a protein structure using its* C_α_* atoms. The connectivity among the *C_α_* 's is expressed in Kirchhoff matrix **Γ** (see Eq. (1)). Two *C_α_* 's are considered to be in contact if their distance falls within a certain cutoff distance. The cutoff distance between a pair of residues is the only parameter in the model and is normally set to be 7 Å to 8 Å. Let **Δr_i_** and **Δr_j_** represent the instantaneous fluctuations from equilibrium positions of residues i and j and* r_ij_* and* r*_0__ ,*ij*_ be the respective instantaneous and equilibrium distances between residues i and j. The Kirchhoff matrix **Γ** is:

	(1)

where *i* and *j* are the indices of the residues and *r_c_* is the cutoff distance.

The simplicity of the Kirchhoff matrix formulation results from the assumption that the fluctuations of each residue are isotropic and Gaussianly distributed along the X, Y and Z directions. The expected value of residue fluctuations, <**Δr_i_**^2^ >, and correlations, <**Δr_i_** ·  **Δr_j_** >, can be easily obtained from the inverse of the Kirchhoff matrix:

	(2)

	(3)

where *k_B_* is the Boltzmann constant and T is the temperature. *γ* is the spring constant. The <**Δr_i_**^2^ > term is directly proportional to the crystallographic B-factors.

**Anisotropic network model.** GNM provides only the magnitudes of residue fluctuations. To study the motions of a protein in more details, especially to determine the directions of the fluctuations, normal mode analysis (NMA) is needed. Traditional NMA is all-atom based and requires a structure to be first energy-minimized before the Hessian matrix and normal modes can be computed, which was rather cumbersome. Even after the energy minimization, the derivation of the Hessian matrix is not easy due to the complicated all-atom potential. In Tirion's pioneering work [18], the energy minimization step was removed and a much simpler Hookean potential was used, and yet it was shown that the low frequency normal modes remained mostly accurate. Since then, the Hookean spring potentials have been favored in most coarse-grained* C*_α_ models [3,19,20]. One of such models is best known as Anisotropic Network Model (ANM) [3] since it has anisotropic, directional information of the fluctuations. The potential in ANM has the simplest harmonic form. Assuming that a given structure is at equilibrium, the Hessian matrix (3N×3N) can be derived analytically from such a potential [3]. The 3N×3N Hessian matrix **H_ANM_** can be repartitioned into N×N super elements and each super element is a 3×3 tensor.

	(4)

where **H_i j_** is the interaction tensor between residues i and j and can be expressed as:

	(5)

Let **H**^+^ be the pseudo inverse of Hessian matrix **H_ANM_.** The mean square fluctuation <**Δr_i_**^2^ > and correlation <**Δr_i_** · **Δr_j_** > can be calculated by summing over the *X*, *Y* and *Z* components:

	(6)

	(7)

**Strengths and limitations of GNM and ANM.** The advantages of ANM or GNM over the conventional NMA lie in several aspects: (i) it is a coarse-grained model and uses the* C_α_*'s to represent the residues in a structure; (ii) it does not require energy minimization and thus can be applied directly to crystal structures to compute the modes of motions; (iii) it provides analytical solutions to the mean square fluctuations and motion correlations.

*The limitations of the GNM model.* GNM provides only information on the magnitudes of residue fluctuations but no directional information. Therefore, the modes of GNM should not be interpreted as protein motions or components of the motions, since the potential in GNM is not rotationally invariant [21].

*The limitations of the ANM model.* In contrast to that in GNM, the potential in ANM is based on simple, harmonic Hookean springs and is rotationally invariant. And thus, the modes of ANM do represent the possible modes of protein motions. In doing this, however, ANM loses a significant amount of precision in predicting the magnitudes of the fluctuations. The reason is that, in GNM, the fluctuations in the separation between a pair of residues are assumed to be Gaussianly distributed and isotropic, while in ANM, because only a Hookean spring is attached between a pair of residues i and j, the fluctuation of residue j is constrained only longitudinally along the axis from i to j. The fluctuation is unconstrained transversely. The interaction spring tensor  between residues i and j in Eq. (5) becomes the following in the local frame (where the Z axis is along the direction from residues i to j):

	(8)

Because the fluctuation of residue j is unconstrained transversely relative to residue i, the fluctuations given by ANM are less realistic than those by GNM, which are assumed to be isotropic. The isotropy in GNM is equivalent to an interaction spring tensor between residues i and j of the following form:

	(9)

From the two tensors  and  given in Eqs. 8 and 9, the causes for the limitations in GNM and ANM are clearly displayed. The unrealistic-ness in ANM is an artifact resulting from its over-simplified potential. The isotropy assumption of GNM, on the other hand, does a better job than ANM in modeling the effect of residue interactions on the magnitudes of the fluctuations, but gives up completely on representing the anisotropic nature that is intrinsic to all physical forces and interactions, since only the magnitudes of the mean-square fluctuations and cross-correlations were of concern when GNM was first proposed. Therefore, to overcome the limitations of GNM and ANM, what is needed is a generalized interaction spring tensor that both is anisotropic and can exert more proper constraints on the fluctuations than the ANM tensor  does. This calls for a model that has a physically more realistic potential than that of ANM. Since potentials with only two-body interactions can provide only longitudinal constraints, it is necessary to include multi-body interactions in the potential in order to have transversal constraints as well. The multi-body interactions provide additional diagonal and off-diagonal terms to the interaction spring tensor between residues i and j. For example, by properly including three-body interactions, the interaction spring tensor may look like:

	(10)

where *k* represent the indices of the residues that interact with both residues i and j through three-body interaction *S*. The first tensor on the right side of the equation represents the two-body interaction, which is similar to , except that the interaction strength *T*(*i*, *j*) depends on residues i and j, and thus may be distance-dependent as well.

**Our contributions.** To overcome the limitations of ANM and GNM, we have developed a generalized spring tensor model for studying protein fluctuation dynamics and conformation changes. It is called generalized spring tensor model, or STeM, for the reason that the interaction between a pair of residues i and j is no longer a Hookean spring that has the tensor form of Eq. (8), but takes a generalized tensor form (similar to that in Eq. 10) that can provide both longitudinal and transversal constraints on a residue's fluctuations relative to its neighbours. We obtain the generalized tensor form by deriving the Hessian matrix from a physically more realistic G*ō*-like potential (Eq. 11), which has been successfully used in many MD simulations to study protein folding processes and conformation changes [22-24]. In additional to the Hookean spring interactions, the potential includes bond bending and torsional interactions, both of which had been found to be helpful in removing the "tip effect" of the ANM model [9]. The inclusion of the bond bending and torsional interactions is reflected in the generalized tensor spring interaction between residues i and j, in such a way that the tensor now includes not only the two-body interaction between residues i and j, but also three-body and four-body interactions that involve residues i and j (see Eq. 10). In doing this, the STeM model is able to integrate all the aforementioned attractive features of ANM and GNM and overcome their limitations. Specifically, STeM performs equally well in B-factors predictions as GNM* and* has the ability to predict the directions of the fluctuations as ANM. This is accomplished with virtually no performance slowdown. The only potential drawback of this model is the significantly increased complexity in deriving the Hessian matrix. Fortunately, this has been done once for all and the derivation results are available electronically at http://www.cs.iastate.edu/~gsong/STeM. STeM is physically more accurate by explicitly including the bond bending and torsional interactions since they capture the chain behavior of protein molecules, which are neglected in most elastic network models where a protein is treated as an elastic rubber. Therefore, we have reasons to expect this model will further distinguish itself in studying protein dynamics where a correct modeling of bond bending and/or torsional rotations is critical.

## Results and discussion

### Crystallographic B-factor prediction

**Figure 1:**
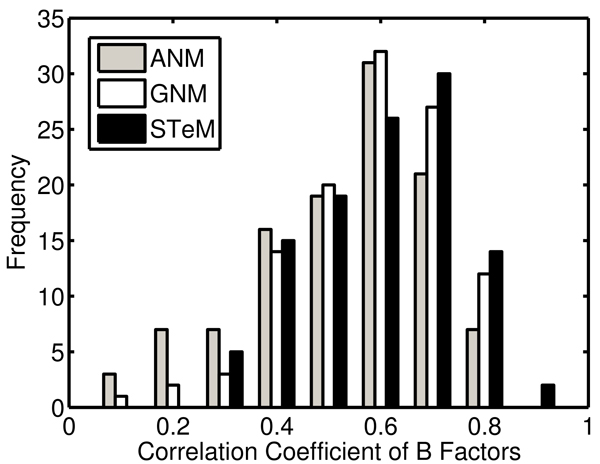
The distributions of the correlation coefficients between the experimental and calculated B-factors

**Figure 2:**
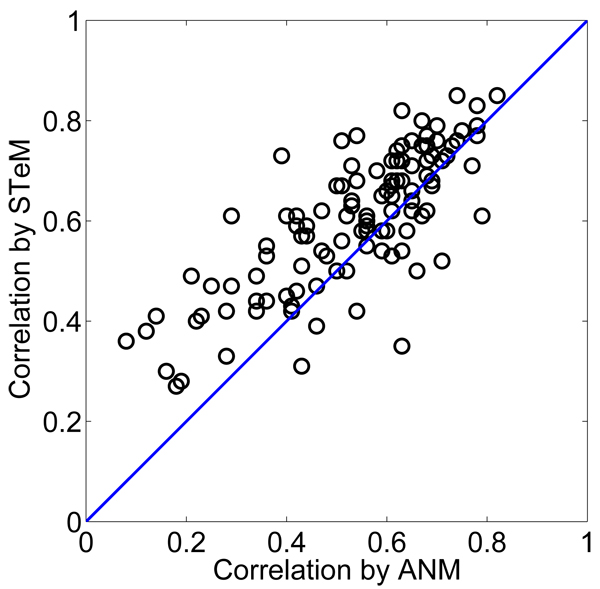
**The scatter plot of the correlation coefficients by ANM and those by STeM** For 80% of the proteins listed in Table 1, STeM does better than ANM.

Table 1 shows the correlation coefficients between the experimental and calculated B-factors of the 111 proteins in the first dataset. The mean values of the correlation coefficients of ANM, GNM, and STeM are 0.53, 0.59, and 0.60 respectively. STeM provides the directional information of the residue fluctuations as ANM and has an accuracy even slightly better than GNM in B-factor predictions. Figure 1 shows the distributions of the correlation coefficients between the calculated B-factors and the experimental B-factors. STeM is the only model in which there are instances where the correlation coefficient is above 0.85 and no instances where the correlation coefficient is below 0.25. This implies that the performance of STeM is more steady than either ANM or GNM. The scatter plot of the correlation coefficients between ANM and STeM in Figure 2 shows that STeM performs better than ANM for 80% of the proteins in the dataset. Protein structures of higher resolution have more accurate data on atom coordinates and B-factors. We investigate whether our model's performance can be further improved when the dataset used is limited to structures with higher resolution. We select the 12 structures with resolution better than 1.3 A from the first dataset. The mean values of the correlation coefficients of these 12 structures are 0.56, 0.62, and 0.63 for ANM, GNM, and STeM, respectively, which gives an improvement of about 5%-6% for all of the three models. Since the improvement is based on a relatively small set of 12 structures, a larger dataset is needed to further examine this potential dependence of B-factor prediction accuracy on structure quality.

**Table 1:** The correlation coefficients between the experimental and calculated B-factors using different models

Protein	R(Å)	ANM	GNM	STeM	Protein	R(Å)	ANM	GNM	STeM	Protein	R(Å)	ANM	GNM	STeM
1AAC	1.31	0.7	0.71	0.76	1ADS	1.65	0.77	0.74	0.71	1AHC	2.00	0.79	0.68	0.61
1AKY	1.63	0.56	0.72	0.6	1AMM	1.20	0.56	0.72	0.55	1AMP	1.80	0.62	0.59	0.68
1ARB	1.20	0.78	0.76	0.83	1ARS	1.80	0.14	0.43	0.41	1ARU	1.60	0.7	0.78	0.79
1BKF	1.60	0.52	0.43	0.5	1BPI	1.09	0.43	0.56	0.57	1CDG	2.00	0.65	0.62	0.71
1CEM	1.65	0.51	0.63	0.76	1CNR	1.05	0.34	0.64	0.42	1CNV	1.65	0.69	0.62	0.68
1CPN	1.80	0.51	0.54	0.56	1CSH	1.65	0.44	0.41	0.57	1CTJ	1.10	0.47	0.39	0.62
1CUS	1.25	0.74	0.66	0.76	1DAD	1.60	0.28	0.5	0.42	1DDT	2.00	0.21	-0.01	0.49
1EDE	1.90	0.67	0.63	0.75	1EZM	1.50	0.56	0.6	0.58	1FNC	2.00	0.29	0.59	0.61
1FRD	1.70	0.54	0.83	0.77	1FUS	1.30	0.4	0.63	0.61	1FXD	1.70	0.58	0.56	0.7
1GIA	2.00	0.68	0.67	0.69	1GKY	2.00	0.36	0.55	0.44	1GOF	1.70	0.75	0.76	0.78
1GPR	1.90	0.65	0.62	0.66	1HFC	1.50	0.63	0.38	0.35	1IAB	1.79	0.36	0.42	0.53
1IAG	2.00	0.34	0.52	0.44	1IFC	1.19	0.61	0.67	0.53	1IGD	1.10	0.18	0.44	0.27
1IRO	1.10	0.82	0.51	0.85	1JBC	1.15	0.72	0.7	0.73	1KNB	1.70	0.63	0.66	0.54
1LAM	1.60	0.53	0.63	0.71	1LCT	2.00	0.52	0.57	0.61	1LIS	1.90	0.16	0.43	0.3
1LIT	1.55	0.65	0.62	0.76	1LST	1.80	0.39	0.72	0.73	1MJC	2.00	0.67	0.67	0.61
1MLA	1.50	0.59	0.57	0.54	1MRJ	1.60	0.66	0.49	0.5	1NAR	1.80	0.62	0.76	0.74
1NFP	1.60	0.23	0.48	0.41	1NIF	1.70	0.42	0.58	0.61	1NPK	1.80	0.53	0.55	0.64
1OMP	1.80	0.61	0.63	0.65	1ONC	1.70	0.55	0.7	0.58	1OSA	1.68	0.36	0.42	0.55
1OYC	2.00	0.78	0.73	0.77	1PBE	1.90	0.53	0.61	0.63	1PDA	1.76	0.6	0.76	0.58
1PHB	1.60	0.56	0.52	0.59	1PHP	1.65	0.59	0.63	0.65	1PII	2.00	0.19	0.44	0.28
1PLC	1.33	0.41	0.47	0.42	1POA	1.50	0.54	0.66	0.42	1POC	2.00	0.46	0.52	0.39
1PPN	1.60	0.61	0.64	0.67	1PTF	1.60	0.47	0.6	0.54	1PTX	1.30	0.65	0.51	0.62
1RA9	2.00	0.48	0.61	0.53	1RCF	1.40	0.59	0.63	0.58	1REC	1.90	0.34	0.5	0.49
1RIE	1.50	0.71	0.25	0.52	1RIS	2.00	0.25	0.24	0.47	1RRO	1.30	0.08	0.31	0.36
1SBP	1.70	0.69	0.72	0.67	1SMD	1.60	0.5	0.62	0.67	1SNC	1.65	0.68	0.71	0.72
1THG	1.80	0.5	0.53	0.5	1TML	1.80	0.64	0.64	0.58	1UBI	1.80	0.56	0.69	0.61
1WHI	1.50	0.12	0.33	0.38	1XIC	1.60	0.29	0.4	0.47	2AYH	1.60	0.63	0.73	0.82
2CBA	1.54	0.67	0.75	0.8	2CMD	1.87	0.68	0.6	0.62	2CPL	1.63	0.61	0.6	0.72
2CTC	1.40	0.63	0.67	0.75	2CY3	1.70	0.51	0.5	0.67	2END	1.45	0.63	0.71	0.68
2ERL	1.00	0.74	0.73	0.85	2HFT	1.69	0.63	0.79	0.72	2IHL	1.40	0.62	0.69	0.72
2MCM	1.50	0.78	0.83	0.79	2MHR	1.30	0.65	0.52	0.64	2MNR	1.90	0.46	0.5	0.47
2PHY	1.40	0.54	0.55	0.68	2RAN	1.89	0.43	0.4	0.31	2RHE	1.60	0.28	0.38	0.33
2RN2	1.48	0.68	0.71	0.75	2SIL	1.60	0.43	0.5	0.51	2TGI	1.80	0.69	0.71	0.73
3CHY	1.66	0.61	0.75	0.68	3COX	1.80	0.71	0.71	0.72	3EBX	1.40	0.22	0.58	0.4
3GRS	1.54	0.44	0.57	0.59	3LZM	1.70	0.6	0.52	0.66	3PTE	1.60	0.68	0.83	0.77
4FGF	1.60	0.41	0.27	0.43	4GCR	1.47	0.73	0.81	0.75	4MT2	2.00	0.42	0.37	0.46
5P21	1.35	0.4	0.51	0.45	7RSA	1.26	0.42	0.63	0.59	8ABP	1.49	0.61	0.82	0.62

Column R(Å) gives the resolution of each structure.

### The contributions of different interaction terms to the fluctuations

The G*ō*-like potential in eq. (11) has four different interaction terms, namely, bond stretching, bond bending, torsional interactions, and the non-bonded interactions. It is of great interest to investigate the relative contributions of these different terms to the agreement with experimental B-factors. Since only the non-bonded interaction term (*V_4_*) is able to provide by itself enough constraints to ensure the Hessian matrix to have no more than six zero eigenvalues,* V_4_* is used as the base term for the evaluation of different terms' contributions to the mean-square fluctuations. The Hessian matrix of ANM, denoted by **H_ANM_**, is used as another baseline for comparison purposes. Table 2 lists the contributions of these different terms to the improvement of B-factor predictions as they are added to the potential.

**Table 2:** The contributions of different interaction terms to the agreement with experimental B-factors

Hessian matrices used	Correlation Coefficient with B-factors	Improvement with respect to ANM
**H_ANM_**	0.53	0.00
	0.55	0.02
	0.57	0.04
	0.57	0.04
	0.56	0.03
	0.59	0.06
	0.58	0.05
	0.57	0.04
	0.60	0.07
	0.54	0.01
	0.54	0.01
	0.54	0.01
	0.56	0.03

**H_ANM_** is the Hessian matrix of ANM. , , , and  are the Hessian matrices of the bond stretching (*V*_1_), bond bending (*V*_2_), torsional rotation (*V*_3_), and non-local interaction (*V*_4_) terms, respectively.

First, it is seen that the non-bonded interactions, as are present in  and** H_ANM_** , play a dominant role in contributing to the B-factors. This is not surprising since the mean-square fluctuations of a residue are mostly constrained by its interactions with its spatial neighbours, most of which are through non-bonded interactions. What is more interesting is that  term alone performs better than **H_ANM_.** This is in agreement with recent results that the performance of B-factor predictions can be improved by using distance-dependent force constants [25,26]. Particularly, the spring constants that take the form of inverse distance square have been shown to be superior in a recent exhaustive study that experimented with different distance-dependent spring constants on a large dataset [10]. The Taylor expansion of the non-bonded interaction term (*V*_4_) shows that it has an equivalent spring constant of the form  (see Eq. 36), which is exactly proportional to the inverse of the pairwise distance square. Thus, STeM provides a physics-based explanation for the choice of using inverse square distance spring constants.

The contribution to the improvement in B-factor predictions from each of the bonded interactions, such as that of bond stretching, is small, as had been pointed out by Bahar et al when GNM was first proposed over a decade ago [2]. However, when the contributions of all of these four terms are added up, they together enable the STeM model to gain a significant improvement over ANM to reach the level of accuracy on a par with GNM.

### Conformational change evaluation

It is known that the modes derived from the open form of a structure have better overlaps and correlations with the direction of a protein's conformation change than the ones derived from the closed form [20]. Here we apply the STeM model to study the conformation changes between the open and closed forms of 20 proteins and the open forms are used to calculate the normal modes. Table 3 lists the overlaps and correlations of the observed conformation changes and the indices of the modes that are most involved in the conformation changes. GNM is not considered since it cannot provide directional information. The mean values of the overlaps and correlation coefficients of ANM are 0.49 and 0.61 respectively, and 0.52 and 0.64 respectively for STeM. These amount to an improvement of about 5% for STeM over ANM on both overlap and correlation. Since the results are obtained based on a relatively small set of 20 protein pairs, the significance of the improvement seen here needs to be further tested by conducting a more exhaustive analysis that uses a larger set of proteins and varying parameters, and preferably taking into account the effect of crystal packing as well. We will leave this for future work. It is also worth noting that, in both the overlap and correlation calculations, the modes that are most involved in the conformation change tend to have lower indices in STeM than in ANM (see Table 3), which may imply the modes of STeM be of higher quality than those of ANM.

**Table 3:** The overlaps and correlations between the observed conformation changes and the most involved modes using different models and the open conformations

Protein	Overlap in ANM	Correlation in ANM	Overlap in STeM	Correlation in STeM
Adenylate kinase	0.49(1)	0.62(1)	0.55(1)	0.63 (1)
Alcohol dehydrogenase	0.69(3)	0.54(9)	0.73 (2)	0.65 (30)
Annexin V	0.33(1)	0.60(32)	0.33 (1)	0.56 (22)
Aspartate aminotransferase	0.56(9)	0.63(9)	0.68 (6)	0.67 (6)
Calmodulin	0.44(5)	0.62 (77)	0.48 (1)	0.62 (16)
Che Y protein	0.46(1)	0.78(12)	0.40(1)	0.74(1)
Citrate synthase	0.48(7)	0.72(26)	0.49(5)	0.63(5)
Dihydrofolate reductase	0.71(1)	0.65(1)	0.73(1)	0.66(1)
Diphtheria toxin	0.43(1)	0.69(2)	0.50(2)	0.73(2)
Enolase	0.31(1)	0.45(34)	0.32(1)	0.49(53)
HIV-1 protease	0.67(1)	0.78 (10)	0.85 (1)	0.90(1)
Immunoglobulin	0.68(3)	0.57(3)	0.66(3)	0.58(3)
Lactoferrin	0.48(1)	0.64(24)	0.48(1)	0.70(36)
LAO binding protein	0.81(1)	0.74(1)	0.87(1)	0.80(1)
Maltodextrin binding protein	0.77(2)	0.66(2)	0.80(2)	0.70(2)
Seryl-tRNA synthetase	0.21(4)	0.59(10)	0.21(4)	0.60(37)
Thymidylate synthase	0.37(4)	0.69(9)	0.44(3)	0.68(9)
Triglyceride lipase	0.35(15)	0.50(25)	0.30(14)	0.56(24)
Triose phosphate isomerase	0.15(38)	0.28(11)	0.14(7)	0.30(8)
Tyrosine phosphatase	0.41(2)	0.57(27)	0.42(1)	0.59(25)

## Conclusions

Protein mean-square fluctuations and conformation changes are two closely related aspects of protein dynamics. However, in the past, two separate groups of models were needed to best explain protein mean-square fluctuations or conformation changes. Specifically, the best models for predicting mean-square fluctuations cannot predict conformation changes, and the models that can predict conformation changes do not have the best performance in predicting mean-square fluctuations. There is thus an obvious gap between the models that work well in predicting one aspect of the dynamics and those in another. Since protein mean-square fluctuations and conformation changes are two closely related dynamic phenomena and share a similar physical origin, we reasoned that models based on a physically more accurate potential should be able to bridge the gap and predict both aspects of the protein dynamics well. Indeed, by using a G*ō*-like potential, we have successfully developed a spring tensor model (STeM) that is able to singly predict well both mean-square fluctuations and conformation changes. Specifically, STeM performs equally well in B-factor predictions as GNM* and* has the ability to predict the directions of fluctuations as ANM.

The new STeM model does come with a cost. As is seen, the derivation process of the Hessian matrix in STeM is much more complex than models using only two-body Hookean potentials, such as those used in ANM. However, the introduced complexity in the potential is necessary in resolving the aforementioned gap that is mainly due to over-simplified potentials and in providing a single, unified model for protein dynamics. Moreover, the derivation process, though more complex, needs to be done only once. Examining the different interaction terms in the G*ō* potential and their contributions to the agreement with experimental B-factors provides further benefits. Along the way, we have discovered a physical explanation for why the distance-dependent, inverse distance square (i.e., ) spring constants perform better than the uniform ones. The van der Waals interaction term in the potential naturally renders inverse distance square spring constants! By including the bond bending and torsional interactions and their contributions to the improvement in B-factor predictions, the STeM model confirms the importance of 3-body and 4-body potentials. The importance of multi-body potentials is made even more evident when their contribution to the interaction spring tensor is examined - the multi-body potentials are shown to be necessary in providing proper constraints on residue fluctuations, even transversely. It is worth noting that the 3-body and 4-body potentials introduced through bond bending and torsional interactions only scratch the surface of the full extensity of the multi-body potentials since bond bending and torsional interactions are restricted to only consecutive residues along the protein chain. The improvement seen here calls for other generalized spring tensor models that have a thorough treatment of the multi-body potentials. Chain breaking, such as that due to missing residues, has a more felt impact on STeM than on ANM or GNM, since the first, the second, and the third terms of the potential used to derive the model are all related to the continuity of the chain. We have not evaluated such impact in the current work but this could be a future research direction and our STeM model would be a proper tool for evaluating the impact of chain breaking on protein motions. STeM does not always outperform ANM in B-factor predictions - it does better than ANM for 80% of the proteins studied. it would be interesting to find out why this is so. Crystal packing has been known to impact significantly the mean-square fluctuations. Therefore, a proper inclusion of the crystal packing effect may further enhance STeM's performance. Since STeM takes into account bond bending and torsional interactions, it is expected that it should further distinguish itself in studying protein dynamics where a correct modeling of bond bending or torsional rotations is critical, such as in predicting the *S*^2^ order parameters of NMR structures.

## Methods

In this section we will show the derivations of the Hessian matrix from a G*ō*-like potential proposed by Clementi et al [22].

### The G*ō*-like potential

The G*ō*-like potential in [22] takes the non-native and native (equilibrium) conformations as input and it can be divided into four terms. The first term of this G*ō*-like potential (defined as* V_1_* for later use) preserves the chain connectivity. The second (*V*_2_) and third terms (*V*_3_) define the bond angle and torsional interactions respectively and the last term (*V*_4_) is the nonlocal interactions. The G*ō*-like potential has the following expression:

	(11)

In Eq. (11), *r* and *r*_0_ represent respectively the instantaneous and equilibrium lengths of the virtual bonds between the* C_α_* atoms of consecutive residues. Similarly, the* θ* (*θ*_o_) and *Φ* (*Φ*_0_) are respectively the instantaneous (equilibrium) virtual bond angles formed by three consecutive residues and the instantaneous (equilibrium) virtual dihedral angles formed by four consecutive residues. The* r_ij_* and* r*_0,_*_ij_* represent respectively the instantaneous and equilibrium distances between two non-consecutive residues i and j. The G*ō*-like potential in Eq. (11) includes several force parameters (*K_r _*,* K_θ _*,  , and *ε*) and the values of these parameters are taken directly from [22] without any tuning. The values of these parameters are:* K_r_* = 100*ε*,* K_θ_* = 20*ε*,  =* ε*,  = 0.5*ε* and *ε* = 0.36.

### Anisotropic fluctuations from the second derivative of the G*ō*-like potential

Similar to ANM, STeM has a 3N×3N Hessian matrix that can be decomposed into N×N super-elements. Each super-element in STeM, **H_i,j_**, is a summation of four 3×3 matrices. The first 3×3 matrix is the contribution from bond stretching. The second and third 3×3 matrices are the contributions from bond bending and torsional rotations respectively. The fourth 3×3 matrix is the contribution from nonlocal contacts.

 	(12)

The Hessian matrix is the second derivative of the overall potential (equation 11). Let us first consider the first term of the G*ō*-like potential and let (*X_i_ ,Y_i_ , Z_i_*) and (*X_j_ , Y_j_ , Z_j_*) be the Cartesian coordinates of two consecutive residues i and j.

	(13)

The first and second partial derivatives of *V*_1_ with respect to the X-direction of residue i are

	(14)

	(15)

We will get similar results for the Y- and Z-directions of residue i. Since we focus only on the equilibrium fluctuations, we can have *r* ≅ *r*^0^ at equilibrium and the first and second partial derivatives of *V*_1_ can be further simplified to the following expressions.

	(16)

	(17)

In a similar way, the second cross-derivatives have the following form:

	(18)

Equations 17 and 18 give the elements of the first 3x3 matrix of the super element** H_ij_** in equation 12. For the diagonal super elements** H_ii_**, equations 17 and 18 are substituted by the following:

	(19)

	(20)

Now let us consider the second term of the potential in Eq. (11) and let (*X_i _, Y_i _, Z_i _*), (*X_j _, Y_j _, Z_j _*) and (*X_k _, Y_k _, Z_k_*) be the Cartesian coordinates of three consecutive residues i, j and k. Suppose* θ* is the virtual bond angle formed by these three consecutive residues. Since the second term of the potential is *V_2_* =* K_θ_* (*θ – θ*_0_ )^2^, the first and second partial derivatives of* V*_2_ are

	(21)

	(22)

Since *θ* equals *θ*_0_ at equilibrium, can be further simplified as

	(23)

Likewise,  becomes

	(24)

Let **p** = (*X_i_* — *X_j _,Y_i_* — *Y_j _*,* Z_i_* — *Z_j_*) and **q** = (*X_k_* —* X_j _, Y_k_* —* Y_j _, Z_k_* —* Z_j _*) and define* G* as the following.

	(25)

The* θ* can be expressed as

	(26)

The partial derivatives of* θ* are

	(27)

	(28)

	(29)

The derivative of* G* is

	(30)

We can also get  and .

	(31)

	(32)

Combined eq (23),(27) and (30), we can get the following formula.

	(33)

Similarly, Combined eq (24),(27), (28), (30) and (31), the second cross-derivative  becomes

	(34)

Following a similar approach, we can get  and  and these second cross-derivatives form the elements of the second 3×3 matrix of the super element **H_ij_** in equation 12.

Due to the complexity of the derivation process of the Hessian matrix for the third (dihedral angle) term of the potential, we omit the derivation process here.The complete derivation is given in Additional File 1.

Finally, let's consider the final (non-local contact) term.

	(35)

A talor expansion will give us the following form.

	(36)

Equation 36 has the same harmonic form as the first term but with a different force constant, so the derivation process is the same as the first term. Therefore, we give only the derivation result here.

	(37)

After combining the Hessian matrices from all four terms, we can calculate the pseudo inverse of the final Hessian matrix **H**. The mean square displacement <**Δr_i_**^2^ > and inter residue correlation <**Δr_i_** ·  **Δr_j_** > can be calculated by summing the elements over the *X*, *Y* and *Z* directions.

	(38)

	(39)

### The protein sets studied

To evaluate the STeM model, we apply it to compute B-factors and to study protein conformation changes and compare the results with those computed from ANM and GNM. For B-factors computations, the protein dataset is from [27] and contains 111 proteins. Two proteins, 1CYO and 5PTP, are removed from the dataset because they no longer exist in the current Protein Data Bank [28]. The proteins in the first dataset all have a resolution that is better than or equal to 2.0 Å. For conformation change studies, the dataset is from [20], which contains 20 pairs of protein structures. Each pair of protein structures have significantly large structure difference from each other.

### Evaluation techniques

We used the same evaluation techniques as have been applied before [20,27]. Specifically, the following three numerical measures are used.

The correlation between the experimental and calculated B-factors

The linear correlation coefficient between the experimental and calculated B-factors is calculated using the following formula.

	(40)

where* x_i_* and *y_i_* are respectively the experimental and calculated B-factors of the* C_α_* atom of residue i and  and  are the mean values. N is the number of residues.

The overlap between the experimental observed conformation changes and the calculated modes

The overlap measures the directional similarity between a conformation change and a calculated mode. The formula for calculating the overlap is

	(41)

where *e_i_* is the relative displacement of residue i in a selected mode *e* and *r_i_* is the conformation displacement of residue i.

The correlation between the experimental observed conformation changes and the calculated modes

The correlation measures the magnitude similarity between a conformation change and a calculated mode. The formula used for calculating the correlation is the same as equation (40), with different meaning for *x_i_* and *y_i_*.

	(42)

where *x_i_* is the magnitude of the displacement of residue i in the conformation change and *y_i_* is the magnitude of the displacement of residue i in the selected mode.  and  are the corresponding mean values.

## List of abbreviations used

ENM: Elastic Network Model; GNM: Gaussian Network Model; ANM: Anisotropic Network Model; STeM: Spring Tensor Model

## Competing interests

The authors declare that they have no competing interests.

## Authors' contributions

Tu-Liang Lin collaborated with Guang Song on the ideas of the Spring Tensor Model and applied the model to the predictions of the B-factors and conformation changes. Gunag Song conceived the idea of using a more sophisticated potential and suggested the G*ō*-like potential. Most of the implementation was done by Tu-Liang Lin under the supervision of Guang Song. Both authors read and approved the manuscript.

## Supplementary Material

Additional file 1 hessian4ThirdTerm.pdf
