# Supplementary material for: Generalized spring tensor models for protein fluctuation dynamics and conformation changes
Source: BMC Struct Biol. 2010 May 17;10(Suppl 1):S3. doi: 10.1186/1472-6807-10-S1-S3 (PMC2873826; doi:10.1186/1472-6807-10-S1-S3)
Supplement: Additional file 1 — hessian4ThirdTerm.pdf [file 1472-6807-10-S1-S3-S1.pdf]

I. Additional File 1: The derivation of the Hessian matrix for the dihedral angle term (V_3)

Let ϕ be the torsional angle between four consecutive residues i, j, k, l and (X_i, Y_i, Z_i), (X_j, Y_j, Z_j), (X_k, Y_k, Z_k), and (X_l, Y_l, Z_l) their coordinates. The third term of G$\bar{o}$-like potential is

$$\begin{aligned} V_3 &= K_\phi^{(1)}(1 - cos(\phi - \phi_0)) + K_\phi^{(3)}(1 - cos3(\phi - \phi_0)) \\ &= K_\phi^{(1)}/2 * (\phi - \phi_0)^2 + K_\phi^{(3)} * 9/2 * (\phi - \phi_0)^2 \\ &= (K_\phi^{(1)}/2 + 9 * K_\phi^{(3)}/2) * (\phi - \phi_0)^2 \end{aligned} \tag{1}$$

Let $K_\phi = K_\phi^{(1)}/2 + 9 * K_\phi^{(3)}/2$. The first derivative of V_3 is

$$\frac{\partial V_3}{\partial X_i} = 2K_\phi(\phi - \phi_0)\frac{\partial \phi}{\partial X_i} \tag{2}$$

The second derivative of V_3 is

$$\frac{\partial^2 V_3}{\partial {X_i}^2} = 2K_\phi(\frac{\partial \phi}{\partial X_i})^2 + 2K_\phi(\phi - \phi_0)\frac{\partial^2 \phi}{\partial {X_i}^2} \tag{3}$$

Since ϕ equals ϕ_0 at equilibrium, $\frac{\partial^2 V_3}{\partial {X_i}^2}$ can be further simplified:

$$\frac{\partial^2 V_3}{\partial {X_i}^2} = 2K_\phi(\frac{\partial \phi}{\partial X_i})^2 \tag{4}$$

We can also get $\frac{\partial^2 V_3}{\partial X_i \partial X_j}$.

$$\frac{\partial^2 V_3}{\partial X_i \partial X_j} = 2K_\phi(\frac{\partial \phi}{\partial X_i})(\frac{\partial \phi}{\partial X_j}) \tag{5}$$

Let $\overrightarrow{a} = (X_j - X_i, Y_j - Y_i, Z_j - Z_i)$, $\overrightarrow{b} = (X_k - X_j, Y_k - Y_j, Z_k - Z_j)$ and $\overrightarrow{c} = (X_l - X_k, Y_l - Y_k, Z_l - Z_k)$. Define $\overrightarrow{v_1} = \overrightarrow{a} \times \overrightarrow{b}$ and $\overrightarrow{v_2} = \overrightarrow{b} \times \overrightarrow{c}$. Define G as the following.

$$G = \frac{(\overrightarrow{v_1}.\overrightarrow{v_2})}{|\overrightarrow{v_1}||\overrightarrow{v_2}|} \tag{6}$$

The ϕ can be expressed as

$$\phi = cos^{-1}(\frac{(\overrightarrow{v_1}.\overrightarrow{v_2})}{|\overrightarrow{v_1}||\overrightarrow{v_2}|}) = cos^{-1}(G) \tag{7}$$

The derivative of ϕ is

$$\frac{\partial \phi}{\partial X_i} = \frac{-1}{\sqrt{1 - G^2}}\frac{\partial G}{\partial X_i} \tag{8}$$

The derivative of G is

$$\begin{aligned} \frac{\partial G}{\partial X_i} &= \frac{\partial}{\partial X_i}\frac{\overrightarrow{v_1}.\overrightarrow{v_2}}{|\overrightarrow{v_1}||\overrightarrow{v_2}|} \\ &= \frac{(\frac{\partial \overrightarrow{v_1}}{\partial X_i}\overrightarrow{v_2} + \frac{\partial \overrightarrow{v_2}}{\partial X_i}\overrightarrow{v_1})|\overrightarrow{v_1}||\overrightarrow{v_2}| - (\overrightarrow{v_1}.\overrightarrow{v_2})(\frac{\partial |\overrightarrow{v_1}|}{\partial X_i}|\overrightarrow{v_2}| + \frac{\partial |\overrightarrow{v_2}|}{\partial X_i}|\overrightarrow{v_1}|))}{(|\overrightarrow{v_1}|\overrightarrow{v_2}|)^2} \end{aligned} \tag{9}$$

We can also get $\frac{\partial G}{\partial X_j}$, $\frac{\partial G}{\partial X_k}$ and $\frac{\partial G}{\partial X_l}$.

$$\frac{\partial G}{\partial X_j} = \frac{(\frac{\partial \overrightarrow{v_1}}{\partial X_j}\overrightarrow{v_2} + \frac{\partial \overrightarrow{v_2}}{\partial X_j}\overrightarrow{v_1})|\overrightarrow{v_1}||\overrightarrow{v_2}| - (\overrightarrow{v_1}.\overrightarrow{v_2})(\frac{\partial |\overrightarrow{v_1}|}{\partial X_j}|\overrightarrow{v_2}| + \frac{\partial |\overrightarrow{v_2}|}{\partial X_j}|\overrightarrow{v_1}|))}{(|\overrightarrow{v_1}|\overrightarrow{v_2}|)^2} \tag{10}$$

$$\frac{\partial G}{\partial X_k} = \frac{(\frac{\partial \overrightarrow{v_1}}{\partial X_k}\overrightarrow{v_2} + \frac{\partial \overrightarrow{v_2}}{\partial X_k}\overrightarrow{v_1})|\overrightarrow{v_1}||\overrightarrow{v_2}| - (\overrightarrow{v_1}.\overrightarrow{v_2})(\frac{\partial |\overrightarrow{v_1}|}{\partial X_k}|\overrightarrow{v_2}| + \frac{\partial |\overrightarrow{v_2}|}{\partial X_k}|\overrightarrow{v_1}|))}{(|\overrightarrow{v_1}|\overrightarrow{v_2}|)^2} \tag{11}$$

$$\frac{\partial G}{\partial X_l} = \frac{(\frac{\partial \overrightarrow{v_1}}{\partial X_l}\overrightarrow{v_2} + \frac{\partial \overrightarrow{v_2}}{\partial X_l}\overrightarrow{v_1})|\overrightarrow{v_1}||\overrightarrow{v_2}| - (\overrightarrow{v_1}.\overrightarrow{v_2})(\frac{\partial |\overrightarrow{v_1}|}{\partial X_l}|\overrightarrow{v_2}| + \frac{\partial |\overrightarrow{v_2}|}{\partial X_l}|\overrightarrow{v_1}|))}{(|\overrightarrow{v_1}|\overrightarrow{v_2}|)^2} \tag{12}$$

The $\frac{\partial\overrightarrow{v_1}}{\partial X_i}$ is

$$\begin{aligned}
\frac{\partial\overrightarrow{v_1}}{\partial X_i} &= \frac{\partial(\overrightarrow{a}\times\overrightarrow{b})}{\partial X_i} \\
&= \frac{\partial\overrightarrow{a}}{\partial X_i}\times\overrightarrow{b}+\overrightarrow{a}\times\frac{\partial\overrightarrow{b}}{\partial X_i} \\
&= \frac{\partial(X_j-X_i,Y_j-Y_i,Z_j-Z_i)}{\partial X_i}\times(X_k-X_j,Y_k-Y_j,Z_k-Z_j)+ \\
&\quad (X_j-X_i,Y_j-Y_i,Z_j-Z_i)\times\frac{\partial(X_k-X_j,Y_k-Y_j,Z_k-Z_j)}{\partial X_i} \\
&= (-1,0,0)\times(X_k-X_j,Y_k-Y_j,Z_k-Z_j)+0 \\
&= (0,Z_k-Z_j,Y_j-Y_k) \qquad (13)
\end{aligned}$$

The $\frac{\partial\overrightarrow{v_1}}{\partial X_j}$ is

$$\begin{aligned}
\frac{\partial\overrightarrow{v_1}}{\partial X_j} &= \frac{\partial(\overrightarrow{a}\times\overrightarrow{b})}{\partial X_j} \\
&= \frac{\partial\overrightarrow{a}}{\partial X_j}\times\overrightarrow{b}+\overrightarrow{a}\times\frac{\partial\overrightarrow{b}}{\partial X_j} \\
&= \frac{\partial(X_j-X_i,Y_j-Y_i,Z_j-Z_i)}{\partial X_j}\times(X_k-X_j,Y_k-Y_j,Z_k-Z_j)+ \\
&\quad (X_j-X_i,Y_j-Y_i,Z_j-Z_i)\times\frac{\partial(X_k-X_j,Y_k-Y_j,Z_k-Z_j)}{\partial X_j} \\
&= (1,0,0)\times(X_k-X_j,Y_k-Y_j,Z_k-Z_j)+(X_j-X_i,Y_j-Y_i,Z_j-Z_i)\times(-1,0,0) \\
&= (0,Z_j-Z_k,Y_k-Y_j)+(0,Zi-Zj,Y_j-Y_i) \\
&= (0,Z_i-Z_k,Y_k-Y_i) \qquad (14)
\end{aligned}$$

The $\frac{\partial\overrightarrow{v_1}}{\partial X_k}$ is

$$\begin{aligned}
\frac{\partial\overrightarrow{v_1}}{\partial X_k} &= \frac{\partial(\overrightarrow{a}\times\overrightarrow{b})}{\partial X_k} \\
&= \frac{\partial\overrightarrow{a}}{\partial X_k}\times\overrightarrow{b}+\overrightarrow{a}\times\frac{\partial\overrightarrow{b}}{\partial X_k} \\
&= \frac{\partial(X_j-X_i,Y_j-Y_i,Z_j-Z_i)}{\partial X_k}\times(X_k-X_j,Y_k-Y_j,Z_k-Z_j)+ \\
&\quad (X_j-X_i,Y_j-Y_i,Z_j-Z_i)\times\frac{\partial(X_k-X_j,Y_k-Y_j,Z_k-Z_j)}{\partial X_k} \\
&= 0+(X_j-X_i,Y_j-Y_i,Z_j-Z_i)\times(1,0,0) \\
&= (0,Z_j-Z_i,Y_i-Y_j) \qquad (15)
\end{aligned}$$

The $\frac{\partial\overrightarrow{v_1}}{\partial X_l}$ is

$$\begin{aligned}
\frac{\partial\overrightarrow{v_1}}{\partial X_l} &= \frac{\partial(\overrightarrow{a}\times\overrightarrow{b})}{\partial X_l} \\
&= \frac{\partial\overrightarrow{a}}{\partial X_l}\times\overrightarrow{b}+\overrightarrow{a}\times\frac{\partial\overrightarrow{b}}{\partial X_l} \\
&= \frac{\partial(X_j-X_i,Y_j-Y_i,Z_j-Z_i)}{\partial X_l}\times(X_k-X_j,Y_k-Y_j,Z_k-Z_j)+ \\
&\quad (X_j-X_i,Y_j-Y_i,Z_j-Z_i)\times\frac{\partial(X_k-X_j,Y_k-Y_j,Z_k-Z_j)}{\partial X_l} \\
&= 0 \qquad (16)
\end{aligned}$$

We can also get $\frac{\partial \overrightarrow{v_2}}{\partial X_i}$, $\frac{\partial \overrightarrow{v_2}}{\partial X_j}$, $\frac{\partial \overrightarrow{v_2}}{\partial X_k}$ and $\frac{\partial \overrightarrow{v_2}}{\partial X_l}$.

$$\frac{\partial \overrightarrow{v_2}}{\partial X_i} = 0 \tag{17}$$

$$\begin{aligned}
\frac{\partial \overrightarrow{v_2}}{\partial X_j} &= \frac{\partial(\overrightarrow{b} \times \overrightarrow{c})}{\partial X_j} \\
&= \frac{\partial \overrightarrow{b}}{\partial X_j} \times \overrightarrow{c} + \overrightarrow{b} \times \frac{\partial \overrightarrow{c}}{\partial X_j} \\
&= \frac{\partial (X_k - X_j, Y_k - Y_j, Z_k - Z_j)}{\partial X_j} \times (X_l - X_k, Y_l - Y_k, Z_l - Z_k) + \\
&\quad (X_k - X_j, Y_k - Y_j, Z_k - Z_j) \times \frac{\partial (X_l - X_k, Y_l - Y_k, Z_l - Z_k)}{\partial X_j} \\
&= (-1, 0, 0) \times (X_l - X_k, Y_l - Y_k, Z_l - Z_k) \\
&= (0, Z_l - Z_k, Y_k - Y_l)
\end{aligned} \tag{18}$$

$$\begin{aligned}
\frac{\partial \overrightarrow{v_2}}{\partial X_k} &= \frac{\partial(\overrightarrow{b} \times \overrightarrow{c})}{\partial X_k} \\
&= \frac{\partial \overrightarrow{b}}{\partial X_k} \times \overrightarrow{c} + \overrightarrow{b} \times \frac{\partial \overrightarrow{c}}{\partial X_k} \\
&= \frac{\partial (X_k - X_j, Y_k - Y_j, Z_k - Z_j)}{\partial X_k} \times (X_l - X_k, Y_l - Y_k, Z_l - Z_k) + \\
&\quad (X_k - X_j, Y_k - Y_j, Z_k - Z_j) \times \frac{\partial (X_l - X_k, Y_l - Y_k, Z_l - Z_k)}{\partial X_k} \\
&= (1, 0, 0) \times (X_l - X_k, Y_l - Y_k, Z_l - Z_k) + (X_k - X_j, Y_k - Y_j, Z_k - Z_j) \times (-1, 0, 0) \qquad (19) \\
&= (0, Zk - Zl, Yl - Yk) + (0, Zj - Zk, Yk - Yj) \\
&= (0, Zj - Zl, Yl - Yj) \qquad (20)
\end{aligned}$$

$$\begin{aligned}
\frac{\partial \overrightarrow{v_2}}{\partial X_l} &= \frac{\partial(\overrightarrow{b} \times \overrightarrow{c})}{\partial X_l} \\
&= \frac{\partial \overrightarrow{b}}{\partial X_l} \times \overrightarrow{c} + \overrightarrow{b} \times \frac{\partial \overrightarrow{c}}{\partial X_l} \\
&= \frac{\partial (X_k - X_j, Y_k - Y_j, Z_k - Z_j)}{\partial X_l} \times (X_l - X_k, Y_l - Y_k, Z_l - Z_k) + \\
&\quad (X_k - X_j, Y_k - Y_j, Z_k - Z_j) \times \frac{\partial (X_l - X_k, Y_l - Y_k, Z_l - Z_k)}{\partial X_l} \\
&= 0 + (X_k - X_j, Y_k - Y_j, Z_k - Z_j) \times (1, 0, 0) \\
&= (0, Zk - Zj, Yj - Yk)
\end{aligned} \tag{21}$$

Let $K_1 = (Y_j - Y_i)(Z_k - Z_j) - (Y_k - Y_j)(Z_j - Z_i)$, $K_2 = (X_j - X_i)(Z_k - Z_j) - (X_k - X_j)(Z_j - Z_i)$ and $K_3 = (X_j - X_i)(Y_k - Y_j) - (X_k - X_j)(Y_j - Y_i)$ The $\frac{\partial |\overrightarrow{v_1}|}{\partial X_i}$ is

$$\begin{aligned}
\frac{\partial |\overrightarrow{v_1}|}{\partial X_i} &= \frac{\partial |\overrightarrow{a} \times \overrightarrow{b}|}{\partial X_i} \\
&= \frac{\partial \sqrt{K_1^2 + K_2^2 + K_3^2}}{\partial X_i} \\
&= \frac{1}{2\sqrt{K_1^2 + K_2^2 + K_3^2}} \left(\frac{2K_1 \partial K_1}{\partial X_i} + \frac{2K_2 \partial K_2}{\partial X_i} + \frac{2K_3 \partial K_3}{\partial X_i}\right)
\end{aligned}$$

$$= \frac{1}{2\sqrt{K_1^2+K_2^2+K_3^2}}\{2K_2(Z_j-Z_k)+2K_3(Y_j-Y_k)\} \quad (22)$$

We can also get $\frac{\partial|\overrightarrow{v_1}|}{\partial X_j}$, $\frac{\partial|\overrightarrow{v_1}|}{\partial X_k}$ and $\frac{\partial|\overrightarrow{v_1}|}{\partial X_l}$.

$$\begin{aligned}
\frac{\partial|\overrightarrow{v_1}|}{\partial X_j} &= \frac{\partial|\overrightarrow{a}\times\overrightarrow{b}|}{\partial X_j} \\
&= \frac{\partial\sqrt{K_1^2+K_2^2+K_3^2}}{\partial X_j} \\
&= \frac{1}{2\sqrt{K_1^2+K_2^2+K_3^2}}(\frac{2K_1\partial K_1}{\partial X_j}+\frac{2K_2\partial K_2}{\partial X_j}+\frac{2K_3\partial K_3}{\partial X_j}) \\
&= \frac{1}{2\sqrt{K_1^2+K_2^2+K_3^2}}[2K_2(Z_k-Z_i)+2K_3(Y_k-Y_i)] \quad (23)
\end{aligned}$$

$$\begin{aligned}
\frac{\partial|\overrightarrow{v_1}|}{\partial X_k} &= \frac{\partial|\overrightarrow{a}\times\overrightarrow{b}|}{\partial X_k} \\
&= \frac{\partial\sqrt{K_1^2+K_2^2+K_3^2}}{\partial X_k} \\
&= \frac{1}{2\sqrt{K_1^2+K_2^2+K_3^2}}(\frac{2K_1\partial K_1}{\partial X_k}+\frac{2K_2\partial K_2}{\partial X_k}+\frac{2K_3\partial K_3}{\partial X_k}) \\
&= \frac{1}{2\sqrt{K_1^2+K_2^2+K_3^2}}[2K_2(Z_i-Z_j)+2K_3(Y_i-Y_j)] \quad (24)
\end{aligned}$$

$$\begin{aligned}
\frac{\partial|\overrightarrow{v_1}|}{\partial X_l} &= \frac{\partial|\overrightarrow{a}\times\overrightarrow{b}|}{\partial X_l} \\
&= \frac{\partial\sqrt{K_1^2+K_2^2+K_3^2}}{\partial X_l} \\
&= \frac{1}{2\sqrt{K_1^2+K_2^2+K_3^2}}(\frac{2K_1\partial K_1}{\partial X_l}+\frac{2K_2\partial K_2}{\partial X_l}+\frac{2K_3\partial K_3}{\partial X_l}) \\
&= 0 \quad (25)
\end{aligned}$$

Let $L_1 = (Y_k-Y_j)(Z_l-Z_k)-(Y_l-Y_k)(Z_k-Z_j)$, $L_2 = (X_k-X_j)(Z_l-Z_k)-(X_l-X_k)(Z_k-Z_j)$ and $L_3 = (X_k-X_j)(Y_l-Y_k)-(X_l-X_k)(Y_k-Y_j)$. The $\frac{\partial|\overrightarrow{v_2}|}{\partial X_i}$ is

$$\begin{aligned}
\frac{\partial|\overrightarrow{v_2}|}{\partial X_i} &= \frac{\partial|\overrightarrow{b}\times\overrightarrow{c}|}{\partial X_i} \\
&= \frac{\partial\sqrt{L_1^2+L_2^2+L_3^2}}{\partial X_i} \\
&= \frac{1}{2\sqrt{L_1^2+L_2^2+L_3^2}}(\frac{2L_1\partial L_1}{\partial X_i}+\frac{2L_2\partial L_2}{\partial X_i}+\frac{2L_3\partial L_3}{\partial X_i}) \\
&= \frac{1}{2\sqrt{L_1^2+L_2^2+L_3^2}}[0+0+0] \\
&= 0 \quad (26)
\end{aligned}$$

We can also get $\frac{\partial|\overrightarrow{v_2}|}{\partial X_j}$, $\frac{\partial|\overrightarrow{v_2}|}{\partial X_k}$ and $\frac{\partial|\overrightarrow{v_2}|}{\partial X_l}$.

$$\begin{aligned}
\frac{\partial|\overrightarrow{v_2}|}{\partial X_j} &= \frac{\partial|\overrightarrow{b}\times\overrightarrow{c}|}{\partial X_j} \\
&= \frac{\partial\sqrt{L_1^2+L_2^2+L_3^2}}{\partial X_j} \\
&= \frac{1}{2\sqrt{L_1^2+L_2^2+L_3^2}}\left(\frac{2L_1\partial L_1}{\partial X_j}+\frac{2L_2\partial L_2}{\partial X_j}+\frac{2L_3\partial L_3}{\partial X_j}\right) \\
&= \frac{1}{2\sqrt{L_1^2+L_2^2+L_3^2}}[2L_2(Z_k-Z_l)+2L_3(Y_k-Y_l)]
\end{aligned} \tag{27}$$

$$\begin{aligned}
\frac{\partial|\overrightarrow{v_2}|}{\partial X_k} &= \frac{\partial|\overrightarrow{b}\times\overrightarrow{c}|}{\partial X_k} \\
&= \frac{\partial\sqrt{L_1^2+L_2^2+L_3^2}}{\partial X_k} \\
&= \frac{1}{2\sqrt{L_1^2+L_2^2+L_3^2}}\left(\frac{2L_1\partial L_1}{\partial X_k}+\frac{2L_2\partial L_2}{\partial X_k}+\frac{2L_3\partial L_3}{\partial X_k}\right) \\
&= \frac{1}{2\sqrt{L_1^2+L_2^2+L_3^2}}[2L_2(Z_l-Z_j)+2L_3(Y_l-Y_j)]
\end{aligned} \tag{28}$$

$$\begin{aligned}
\frac{\partial|\overrightarrow{v_2}|}{\partial X_l} &= \frac{\partial|\overrightarrow{b}\times\overrightarrow{c}|}{\partial X_l} \\
&= \frac{\partial\sqrt{L_1^2+L_2^2+L_3^2}}{\partial X_l} \\
&= \frac{1}{2\sqrt{L_1^2+L_2^2+L_3^2}}\left(\frac{2L_1\partial L_1}{\partial X_l}+\frac{2L_2\partial L_2}{\partial X_l}+\frac{2L_3\partial L_3}{\partial X_l}\right) \\
&= \frac{1}{2\sqrt{L_1^2+L_2^2+L_3^2}}[2L_2(Z_j-Z_k)+2L_3(Y_j-Y_k)]
\end{aligned} \tag{29}$$

Combined eq (4),(8), (9), (13), (17), (22) and (26), we can get the following formula.

$$\begin{aligned}
\frac{\partial^2 V_3}{\partial {X_i}^2} &= \frac{2K_\phi}{1-G^2}\left(\frac{(\frac{\partial\overrightarrow{v_1}}{\partial X_i}\overrightarrow{v_2}+\frac{\partial\overrightarrow{v_2}}{\partial X_i}\overrightarrow{v_1})|\overrightarrow{v_1}||\overrightarrow{v_2}|-(\overrightarrow{v_1}.\overrightarrow{v_2})(\frac{\partial|\overrightarrow{v_1}|}{\partial X_i}|\overrightarrow{v_2}|+\frac{\partial|\overrightarrow{v_2}|}{\partial X_i}|\overrightarrow{v_1}|)}{(|\overrightarrow{v_1}|\overrightarrow{v_2}|)^2}\right)^2 \\
&= \frac{2K_\phi}{1-G^2}\left(\frac{(\frac{\partial\overrightarrow{v_1}}{\partial X_i}\overrightarrow{v_2})|\overrightarrow{v_1}||\overrightarrow{v_2}|-(\overrightarrow{v_1}.\overrightarrow{v_2})(\frac{\partial|\overrightarrow{v_1}|}{\partial X_i}|\overrightarrow{v_2}|)}{(|\overrightarrow{v_1}|\overrightarrow{v_2}|)^2}\right)^2 \\
&= \frac{2K_\phi}{1-G^2}\left(\frac{(0,Z_j-Z_k,Y_j-Y_k).\overrightarrow{v_2}|\overrightarrow{v_1}||\overrightarrow{v_2}|-\frac{\overrightarrow{v_1}.\overrightarrow{v_2}}{\sqrt{K_1^2+K_2^2+K_3^2}}[K_2(Z_j-Z_k)+K_3(Y_j-Y_k)]|\overrightarrow{v_2}|}{(|\overrightarrow{v_1}|\overrightarrow{v_2}|)^2}\right)^2
\end{aligned} \tag{30}$$

We can also get $\frac{\partial^2 V_3}{\partial X_i \partial X_j}$:

$$\begin{aligned}
\frac{\partial^2 V_3}{\partial X_i\partial X_j} &= \frac{2K_\phi}{1-G^2}\left(\frac{(\frac{\partial\overrightarrow{v_1}}{\partial X_i}\overrightarrow{v_2}+\frac{\partial\overrightarrow{v_2}}{\partial X_i}\overrightarrow{v_1})|\overrightarrow{v_1}||\overrightarrow{v_2}|-(\overrightarrow{v_1}.\overrightarrow{v_2})(\frac{\partial|\overrightarrow{v_1}|}{\partial X_i}|\overrightarrow{v_2}|+\frac{\partial|\overrightarrow{v_2}|}{\partial X_i}|\overrightarrow{v_1}|)}{(|\overrightarrow{v_1}|\overrightarrow{v_2}|)^2}\right)* \\
&\quad \left(\frac{(\frac{\partial\overrightarrow{v_1}}{\partial X_j}\overrightarrow{v_2}+\frac{\partial\overrightarrow{v_2}}{\partial X_j}\overrightarrow{v_1})|\overrightarrow{v_1}||\overrightarrow{v_2}|-(\overrightarrow{v_1}.\overrightarrow{v_2})(\frac{\partial|\overrightarrow{v_1}|}{\partial X_j}|\overrightarrow{v_2}|+\frac{\partial|\overrightarrow{v_2}|}{\partial X_j}|\overrightarrow{v_1}|)}{(|\overrightarrow{v_1}|\overrightarrow{v_2}|)^2}\right)
\end{aligned}$$

$$
\begin{aligned}
= \quad & \frac{2K_\phi}{1-G^2}\left(\frac{(0, Z_j - Z_k, Y_j - Y_k).\overrightarrow{v_2}|\overrightarrow{v_1}||\overrightarrow{v_2}| - \frac{\overrightarrow{v_1}.\overrightarrow{v_2}}{\sqrt{K_1^2+K_2^2+K_3^2}}[K_2(Z_j - Z_k) + K_3(Y_j - Y_k)]|\overrightarrow{v_2}|}{(|\overrightarrow{v_1}|\overrightarrow{v_2}|)^2}\right) * \\
& [\frac{(0, Z_i + Z_k - 2Z_j, Y_i + Y_k - 2Y_j).\overrightarrow{v_2}|\overrightarrow{v_1}||\overrightarrow{v_2}|}{(|\overrightarrow{v_1}|\overrightarrow{v_2}|)^2}) - \\
& \frac{\frac{\overrightarrow{v_1}.\overrightarrow{v_2}}{\sqrt{K_1^2+K_2^2+K_3^2}}\{[K_2(Z_k - Z_i) + K_3(Y_k - Y_i)]|\overrightarrow{v_2}|\}}{(|\overrightarrow{v_1}|\overrightarrow{v_2}|)^2} - \\
& \frac{\frac{\overrightarrow{v_1}.\overrightarrow{v_2}}{\sqrt{L_1^2+L_2^2+L_3^2}}\{[L_2(Z_k - Z_l) + L_3(Y_k - Y_l)]|\overrightarrow{v_1}|\}}{(|\overrightarrow{v_1}|\overrightarrow{v_2}|)^2}]
\end{aligned} \tag{31}
$$
